# Sonographic sling position and cure rate 10-years after TVT- O procedure

**DOI:** 10.1371/journal.pone.0209668

**Published:** 2019-01-07

**Authors:** Ayman Tamma, Vesna Bjelic-Radisic, Susanne Hölbfer, Gerda Trutnovsky, Karl Tamussino, Thomas Aigmüller, Daniela Ulrich

**Affiliations:** 1 Department of Obstetrics and Gynecology, Medical University of Graz, Graz, Austria; 2 Department of Obstetrics and Gynecology, Wilhelminen Hospital, Vienna, Austria; University Medical Center Utrecht, NETHERLANDS

## Abstract

**Aim:**

To examine the position of the TVT-O sling 10 years postoperatively and its association with outcome.

**Methods:**

A total of 124 patients who received a TVT-O sling at two centers in 2004 and 2007 were invited for follow-up. The position of the sling on perineal ultrasound was described relative to the bladder neck and the lower margin of the pubic symphysis at rest and on Valsalva. Objective cure was defined as a negative cough stress test at 300 ml. Subjective cure was evaluated with the Kings´ Health Questionnaire (KHQ), Incontinence Outcome Questionnaire (IOQ), Female Sexual Function Index Questionnaire (FSFI) and the Patient Global Impression of Improvement score (PGII).

**Results:**

78 of 124 patients (57%) were available for follow-up 10 years after surgery. I Eleven (14%) had undergone reoperation and were excluded. Tapes were visualized in the remaining 67 (54%) women. The subjective and objective cure rates in this sub-cohort were 67% (45/67) and 77% (52/67), respectively. In these 67 women the mean distances from the bladder neck to the proximal edge of the tape (BNTD) during Valsalva maneuver were significantly higher in cured women compared to the not-cured women (11.2 vs. 9.4mm). The distance between tape and urethra (TUD) was significantly lower in cured vs. not cured patients (2.6 vs. 4.1mm). All women with a TUD of >5mm (n = 5) were incontinent. Tape position was not associated with overactive bladder symptoms.

**Conclusions:**

Tape position near the bladder neck and large distance to the urethra is associated with incontinence 10 years after TVT-O.

## Introduction

Stress urinary incontinence (SUI) affects up to 25% of women and can severely impact quality of life (QoL)[1,2] The lifetime risk of SUI surgery for women in the United States has recently been estimated as 13.6%[3], and the number of procedures to correct urinary incontinence have increased during the last decades [4].Although pelvic floor muscle therapy is recommended as first line therapy of SUI, it has been shown to be inferior to midurethral tape surgery, which is the mainstay of surgical treatment for SUI [5].

Midurethral tapes can be placed in a retropubic or transobturator position. While transobturator tapes have a higher risk of repeat surgery, retropubic tapes are associated with higher incidence of bladder injuries and postoperative voiding difficulties [6,7]. Overall, both tapes have high short-term and long-term success rates [7–13].

Perineal or introital sonography can readily image suburethral tapes and may provide insights into the mechanisms of successful or unsuccessful outcomes [14–16]. To date, however, no studies have addressed sonography findings in long-term follow-up studies of patients after midurethral tape surgery. The aim of the present study was to correlate tape position 10 years after TVT-O with objective cure. The study also addressed subjective outcome, QoL, patient satisfaction, and sexual health.

## Methods

This cohort study was performed between 2014 and 2017 in patients who were operated between 2004 and 2007 with the TVT-O (Gynecare, Ethicon, Johnson & Johnson) at two urogynecology units. Preoperative evaluation included a relevant history and clinical examination as well as urodynamic studies with a cough stress test at 300 ml. The TVT-O was inserted according to the original description by de Leval [17]; all surgeons were experienced gynecologists with a major interest in urogynecology. The follow-up examination was performed by five assessors who had not operated the women themselves. All patients had been entered in the Austrian Transobturator Tape Registry [13] and clinical data of some of the patients have been reported in a long-term follow-up study [10]. The study protocol was approved by the local ethical committees of the participating institutions and all patients gave written consent.

The follow-up examination included a relevant history and evaluation of subjective and objective outcomes. Subjective outcome was assessed with standardized and validated questionnaires (QoL was assessed using the Kings health Questionnaire (KHQ) [18] and the incontinence outcome questionnaire (IOQ) [19]; subjective improvement with the Patient Global Impression of Improvement questionnaire (PGI-I) [20]; and sexual health with the Female Sexual Function Index (FSFI) [21]. PGII was successful when ‘‘much better”, ‘‘very much better”, or “a little better” (1–3 on questionnaire) was ticked.

Objective outcome was assessed with clinical examination, urodynamics including cystometry to 300 ml and a cough stress test, perineal sonography, and cystoscopy in case of overactive bladder (OAB) symptoms.

**Fig 1:**
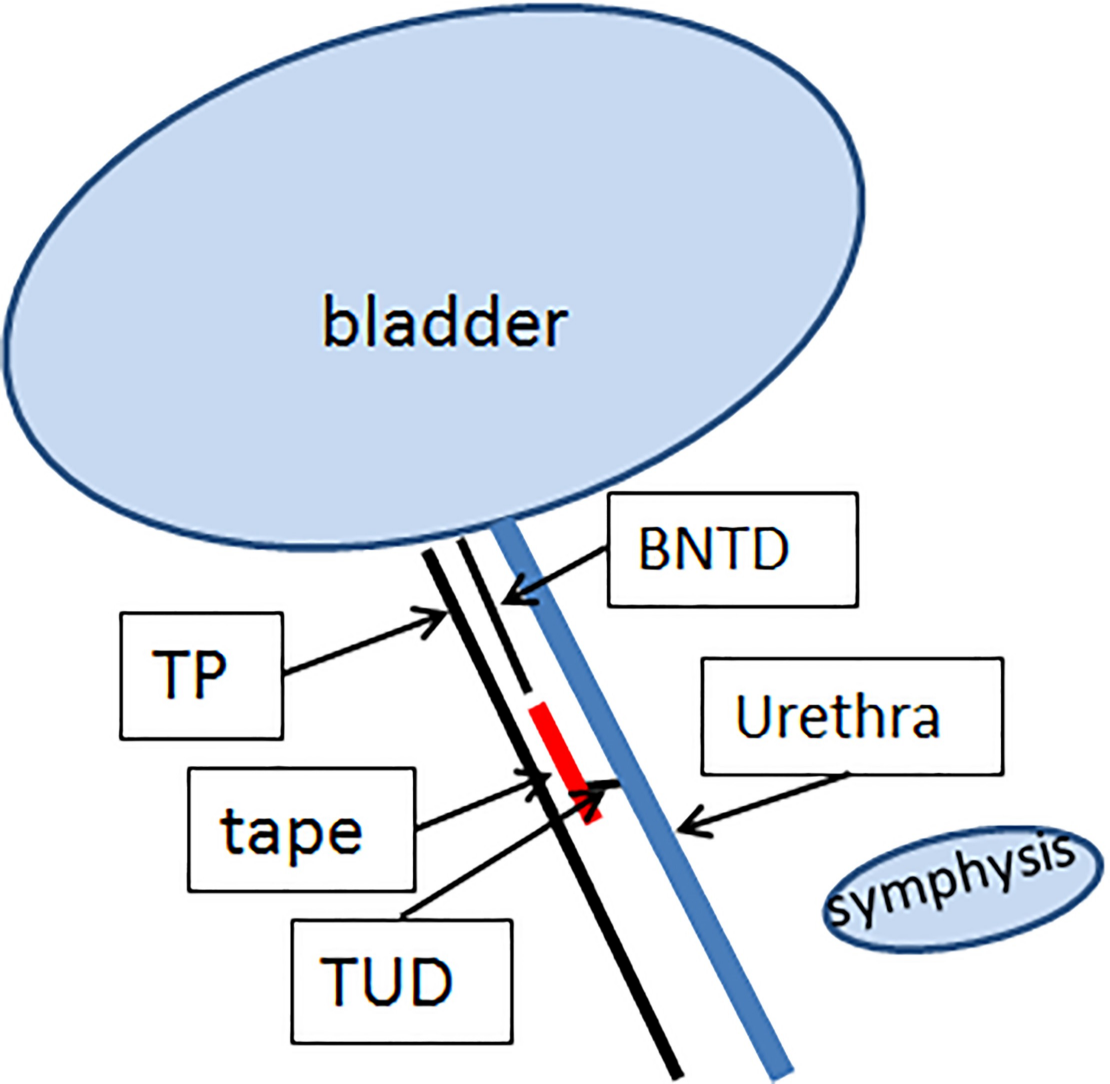
The distance from the posterior side of the urethra at the bladder neck to the nearest, proximal edge of the tape was defined as the “bladder neck to tape distance” (BNTD) BNTD Bladder neck to tape distance; TP Tape percentile; TUD Tape-urethra distance.

**Fig 2:**
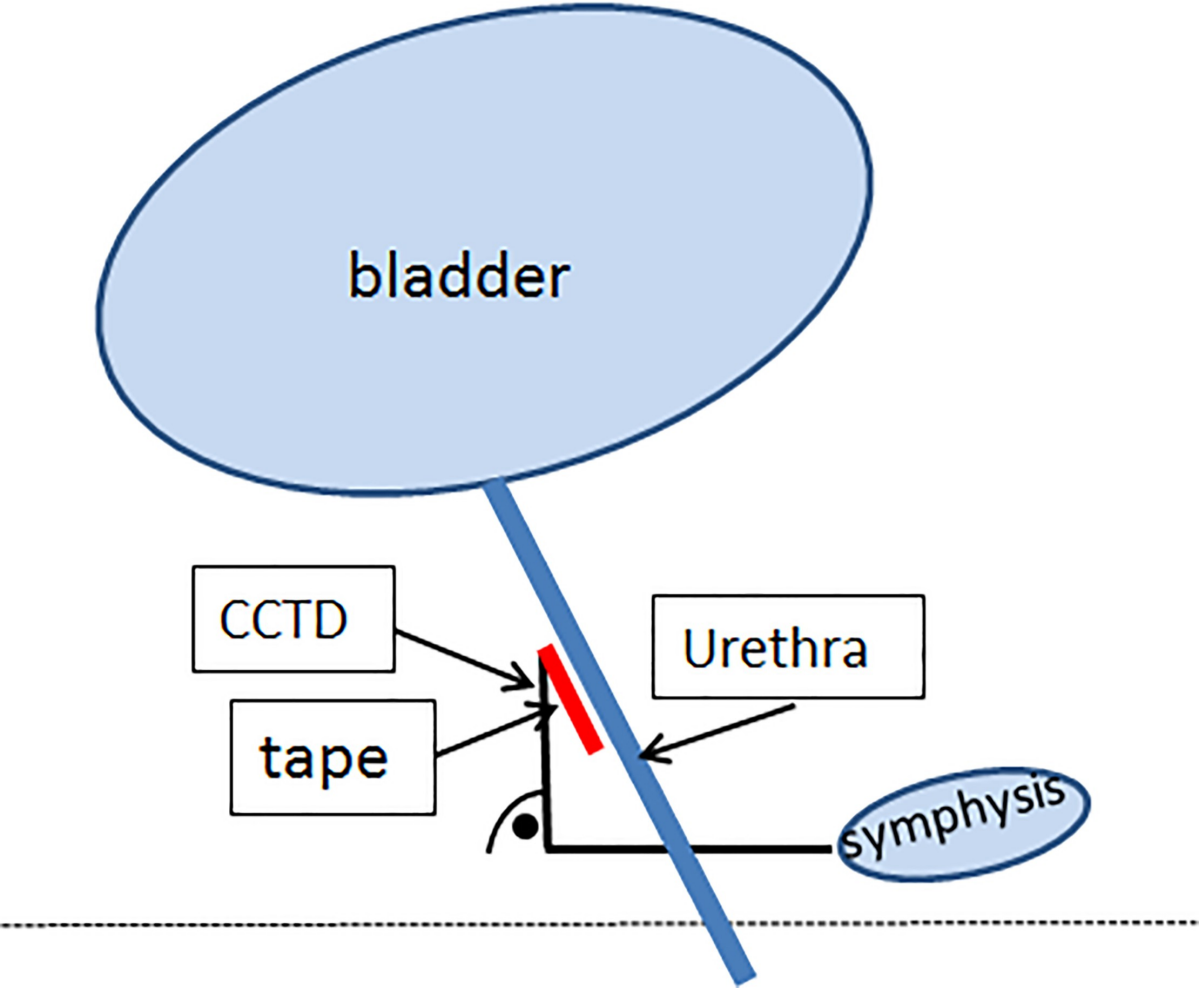
The position of the tape in relation to the lower level of the pubic symphysis was expressed as the “craniocaudal tape distance” (CCTD) CCTD Craniocaudal tape distance.

Perineal ultrasound examination was done to evaluate tape position and functionality with a Siemens Acuson X 300 or a Flex Focus 500 Ultrasound system (BK Ultrasound, Herlev, Denmark). Patients were examined after emptying the bladder in a semisupine position with the probe placed on the labia minora in a sagittal plane. Images were taken in the median sagittal plane and included the pubic symphysis, urinary bladder and urethra [14,15]. The suburethral tape was identified as a hyperechoic structure and the position of the tape was measured in relation to the urethra and in relation to the pubic symphysis, both at rest and during Valsalva. The distance from the posterior side of the urethra at the bladder neck to the nearest, proximal edge of the tape was defined as the “bladder neck to tape distance” (BNTD) as previously described [22] (Fig 1). The position of the tape in relation to the lower level of the pubic symphysis was expressed as the “craniocaudal tape distance” (CCTD) and was measured from the proximal edge of the tape right angular to a horizontal line through the posterior inferior margin of the pubic symphysis (Fig 2). Tape position along the urethra was measured as a percentage of the urethral length, referred to as ‘tape percentile’ and was calculated as follows: the proximal urethral length (distance from bladder neck to proximal point of tape) divided by total urethral length (distance from bladder neck to external urethral meatus) in the sagittal plane, with the bladder neck and the external urethral meatus representing 0% and 100% of the urethral length (Fig 1). The shortest distance between the tape and the hypoechoic structure of the urethra is defined as tape-urethra distance (TUD) [14] (Fig 1).

All women were asked about bladder emptying problems, persisting groin pain or de novo overactive bladder symptoms. Subjective cure was defined as an answer of “never” to the question “Does urine leak when you are physically active, exert yourself, cough, or sneeze?”. Objective cure was defined as a negative cough stress test at a bladder filling of 300 ml.

### Statistical analysis

Differences between cured and not cured patients were analyzed with the chi square test for categorical variables and the U-test for numerical variables. Cohen's kappa was computed to evaluate agreement between objective and subjective cure. The McNemar test was used to test for differences in overactive bladder between pre and post operation. A p- value of < 0.05 was regarded as statistically significant. An estimation of sample size demonstrated that 67 women were required to demonstrate an effect size (d) of 0.8, with 80% power and two-sided to detect significant differences for the TUD between cured and non cured woman. Thus, assuming an eventual dropout rate of 15%, recruitment of 78 women was planned.

## Results

After a follow-up of 10 years 78 of 124 women (57%) after the TVT-O operation were available for the follow-up study including ultrasound examination. Clinical data of 55 of these patients have been reported previously [8]. The mean age at surgery was 58 years (range, 37–80). Eleven of 78 patients (14%) had undergone reoperation for recurrent or persistent SUI or voiding dysfunction in the interim and were excluded from this analysis because correct ultrasound evaluation due to tape removal or placement of a second tape was not possible. Thus, the present study is based on 67 patients.

Demographic data including previous surgery and concomitant procedures are listed in Table 1. Demographic and clinical data at time of follow-up are shown in Table 2. In the study population of patients without tape-specific reoperations the objective cure rate defined as a negative cough stress test at a bladder filling of 300 ml. was 77% (52/67) and the subjective cure rate was 67% (45/67). (Subjective cure was defined as an answer of “never” to the question “Does urine leak when you are physically active, exert yourself, cough, or sneeze?”.) In 87% of the cases, the subjective cure was in accordance with the objective cure (Coefficient of agreement, Cohen's kappa = 0.67, p < .001). 66% (44/67) of women had both subjective and objective cure, one patient had subjective but not objective cure, and 12% (8/67) had objective but not subjective cure. According to the PGI-I, 77% (52/67) considered themselves improved or cured, 9% (6/67) unchanged, and 14% (9/67) worse. Pad usage was 42%, however 9 (13%) women only used one pad a day because they feel more secure with it. Three of 67 patients (4.5%) were found to have vaginal tape exposures, all of which were small and asymptomatic and did not require surgical treatment.

**Table 1:** Demographic data of patients at time of TVT-O surgery (n = 67).

Age (years)	58 ± 10
BMI (kg/m^2^)	29 ± 5
Hormonal status	
Premenopausal	3 (4)
Menopausal	55 (82)
HRT	9 (13)
Smoking	12 (18)
Parity, median (range)	2 (0–5)
Maximal urethral closure pressure (cm H_2_O)	48 ± 26
Previous surgery	
Hysterectomy	20 (30)
POP surgery	5 (7)
Anti-incontinence procedures (Burch)	4 (6)
Concomitant surgeries	
Vaginal hysterectomy	4 (6)
Vaginal hysterectomy + colporrhaphy	7 (10)
Colporrhaphy only	3 (4)
Mesh (vaginal)	1 (1.5)

Data are expressed as number (%) or mean (±SD). HRT = hormonal replacement therapy; UTI = urinary tract infections; POP = pelvic organ prolapse.

**Table 2:** Clinical assessment 10 Years after TVT-O surgery (n = 67).

	N (%)
Smoker	11 (17)
UTI within 1 year	11 (17)
Pad usage	28 (42)
Hormonal status	
Premenopausal	3 (4)
Postmenopausal, with HRT	10 (15)
Postmenopausal, without HRT	54 (81)
Objective cure (Stress test)	
Yes	52 (77)
No	15 (23)
Subjective cure	
Yes	45 (70)
No	22 (30)

Data are expressed as absolute number (%).

**Fig 3:**
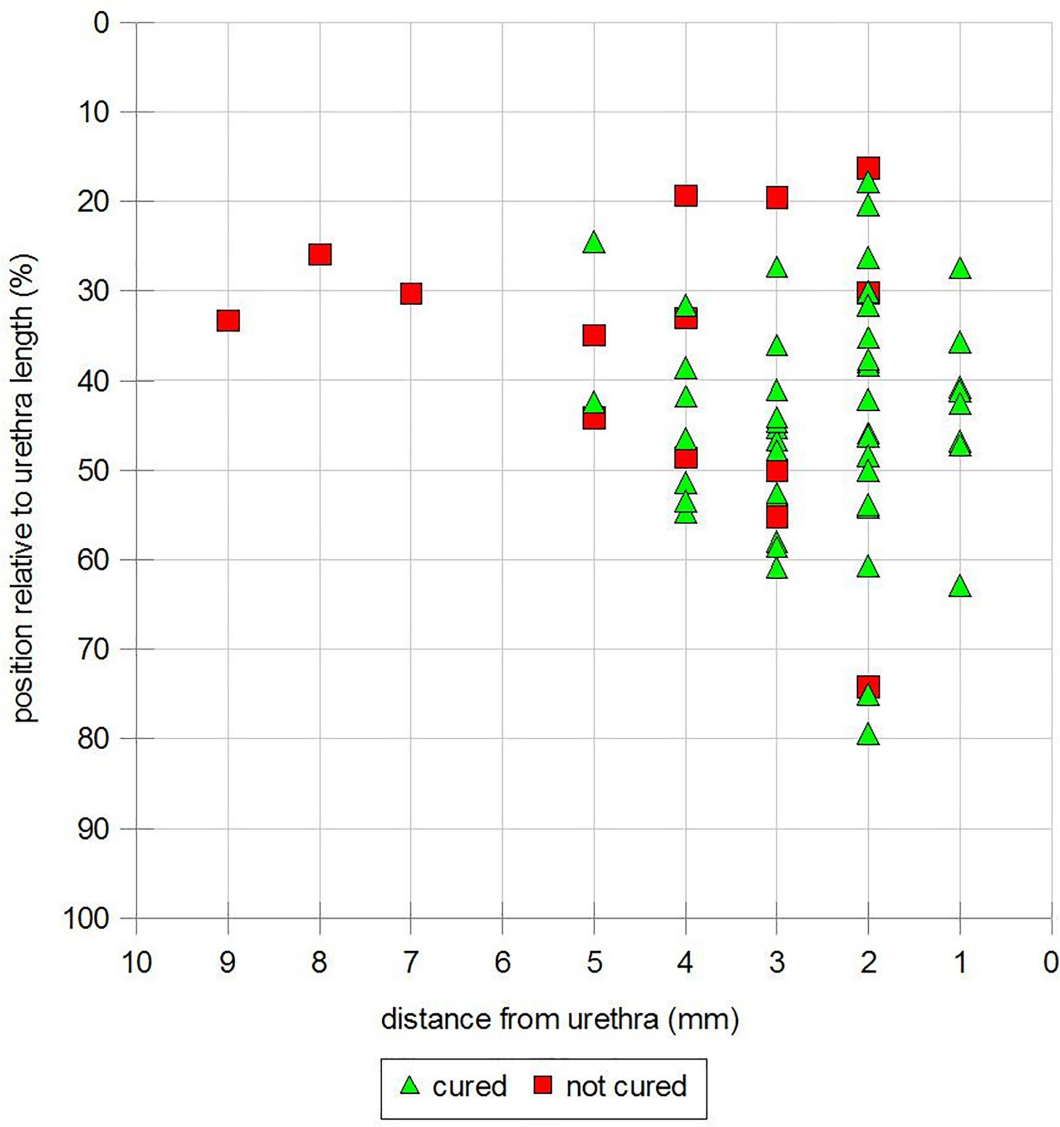
Cured vs. none-cured women according to the stress cough test dependent on tape position relative to TUD and TP.

Tapes were readily visualized in all 67 patients. Ultrasound parameters of the relation of the tape to the urethra and the lower level of the symphysis correlated between objectively and subjectively cured vs. non cured women (Table 3, Fig 3). The distance from the bladder neck to the proximal edge of the tape (BNTD) ranged from 4 to 26 mm at rest and from 1 to 35 mm on Valsalva. No significant differences were seen between subjectively cured vs non cured women, however distance means were significantly higher in the objectively cured vs not cured women on Valsalva (p = 0.021). When the same BNTD was calculated in regard to the total urethral length similar results were obtained; at rest, mean “tape percentile” (TP), percentage relative to the urethral length) was 44% in the subjectively cured group vs 41% in the subjective failure group without significant differences at rest or on Valsalva. In the objectively cured group tape percentile was significantly higher (45%) compared to the not cured group (37%) (p = 0.046). On Valsalva, tape percentiles showed similar values, but without any significant differences between the groups. Due to a high range of tape percentiles there is no cut-off for subjective or objective cure.

**Table 3:** Results of ultrasound examination at 10 year follow-up. Comparison of women with subjective and objective cure (n = 67).

	Subjective cure (SUI)			Objective cure (SUI)		
	Cured	Not cured	p- Value	Cured	Not cured	p- Value
BNTD rest	13.38 ± 4.23	13.33 ± 6.41	.754	13.89 ± 4.60	11.54 ± 6.00	.078
BNTD Valsalva	10.53 ± 6.55	11.35 ± 7.40	0.587	11.17 ± 6.52	9.44 ± 7.74	0.021
CCTD rest	9.17 ± 8.96	10.15 ± 9.25	.762	8.77 ± 9.25	12.14 ± 7.70	.355
CCTD Valsalva	4.74 ± 10.80	4.21 ± 11.07	.940	4.09 ± 10.97	6.45 ± 10.27	.470
TP rest	43.82 ± 13.11	41.10 ± 14.32	.453	44.61 ± 12.32	36.80 ± 16.08	.046
TP Valsalva	40.75 ± 17.23	38.11 ± 14.75	.537	41.46 ± 16.20	34.54 ± 16.53	.130
TUD rest	2.57 ± 0.92	3.58 ± 2.19	0.19	2.57 ± 1.03	4.05 ± 2.28	0.19
TUD Valsalva	2.27 ± 0.88	3.28 ± 1.80	.036	2.33 ± 0.99	3.49 ± 1.89	.033

Data are expressed as median ± standard deviation except for the p-values. BNTD, bladder neck tape distance; CCTD, cranio-caudal tape distance; TP, tape percentile; TUD, tape urethral distance

The absolute distance between tape and urethra (TUD) ranged from 1 to 9 mm at rest and from 0.5 to 7.2 mm on Valsalva. The mean difference at rest was 2.6 ± 0.9 mm in the subjectively cured group and 2.6 ± 1.0 mm in the objectively cured group. This was significantly less (p = 0.19) compared to the non-cured groups (3.58 ± 2.19, 4.05 ± 2.28, respectively). Similar results were obtained on Valsalva with both the subjectively and objectively cured women having lower TUDs than the non-cured women (p = 0.36 and 0.33, respectively).

No significant differences between cured and non-cured women were seen in measurements from the lower level of the symphysis pubis. CCTD varied markedly from 30 mm above to 13 mm below (at rest) and between 14 mm above to 34 mm below the symphysis (on Valsalva).

**Fig 4:**
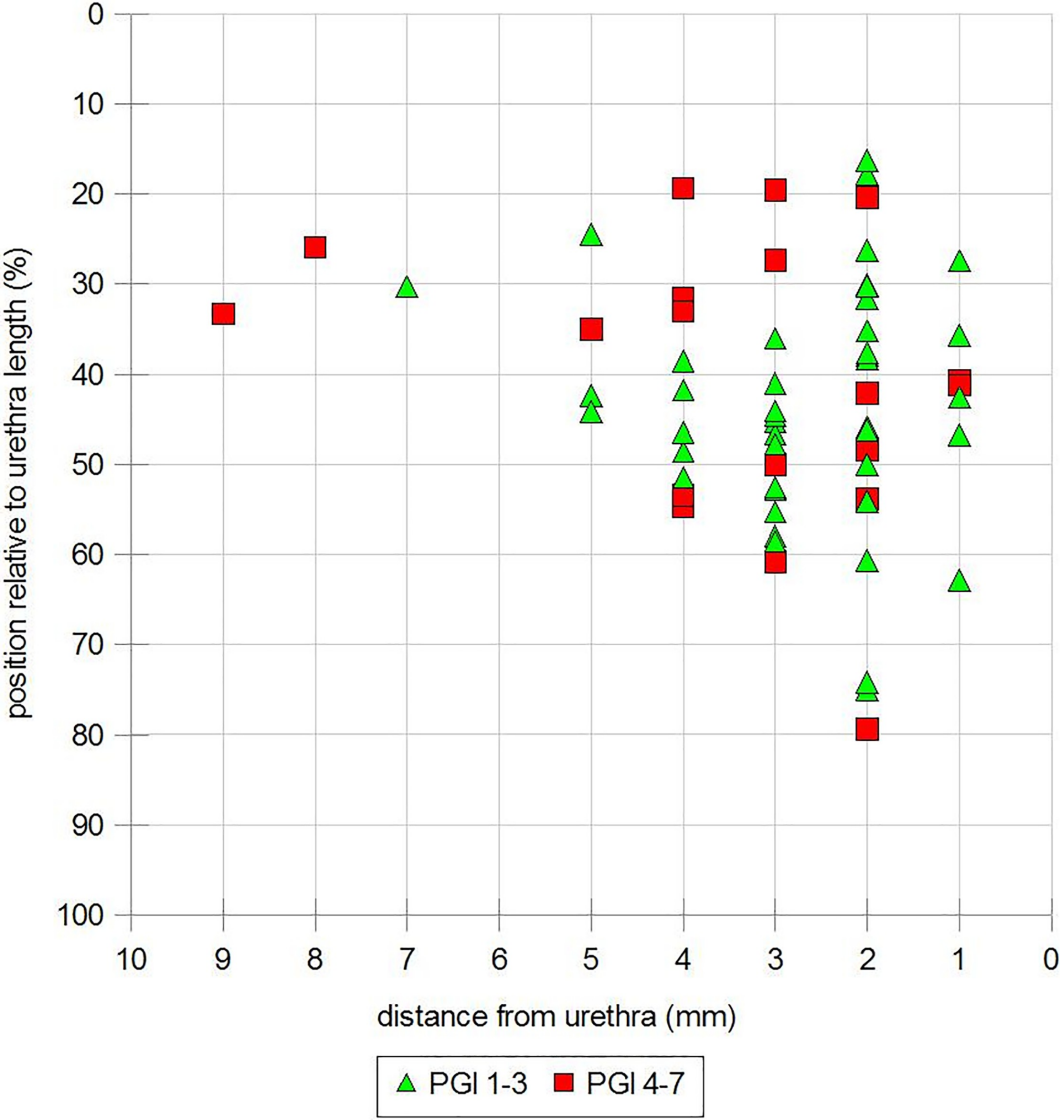
Cured vs. none-cured women according to the PGI-I dependent on tape position relative to TUD and TP.

We further calculated differences between women with or without success according to the PGI-I. No significant differences were seen for any ultrasound parameters between the two groups (Table 4, Fig 4).

**Table 4:** Results of ultrasound examination at 10 year follow-up. Comparison of women with success vs. failure according to the PGI-I (n = 67).

	PGI-I 1–3	PGI-I 4–7	p- Value
BNTD rest	13.57 ± 4.92	13.03 ± 5.39	.661
BNTD Valsalva	10.45 ± 7.04	11.75 ± 6.32	.751
CCTD rest	9.45 ± 8.73	9.58 ± 10.07	.840
CCTD Valsalva	4.90 ± 10.61	4.28 ± 11.67	.862
TP rest	43.87 ± 12.54	40.53 ± 15.85	.320
TP Valsalva	39.87 ± 16.15	40.13 ± 18.01	.879
TUD rest	2.73 ± 1.23	3.40 ± 2.04	.269
TUD Valsalva	2.39 ± 1.18	3.11 ± 1.58	.137

Data are expressed as median ± standard deviation except for the p-values. BNTD, bladder neck tape distance; CCTD, cranio-caudal tape distance; TP, tape percentile; TUD, tape urethral

We attempted to calculate cut-off values as predictors for subjective or objective cure for TP and TUD. Subjectively and objectively cured women had TP in the middle third of the urethra, but no cut-off values could be determined for cure. In contrast, women (n = 5) with a TUD of more than 5mm were incontinent.

**Fig 5:**
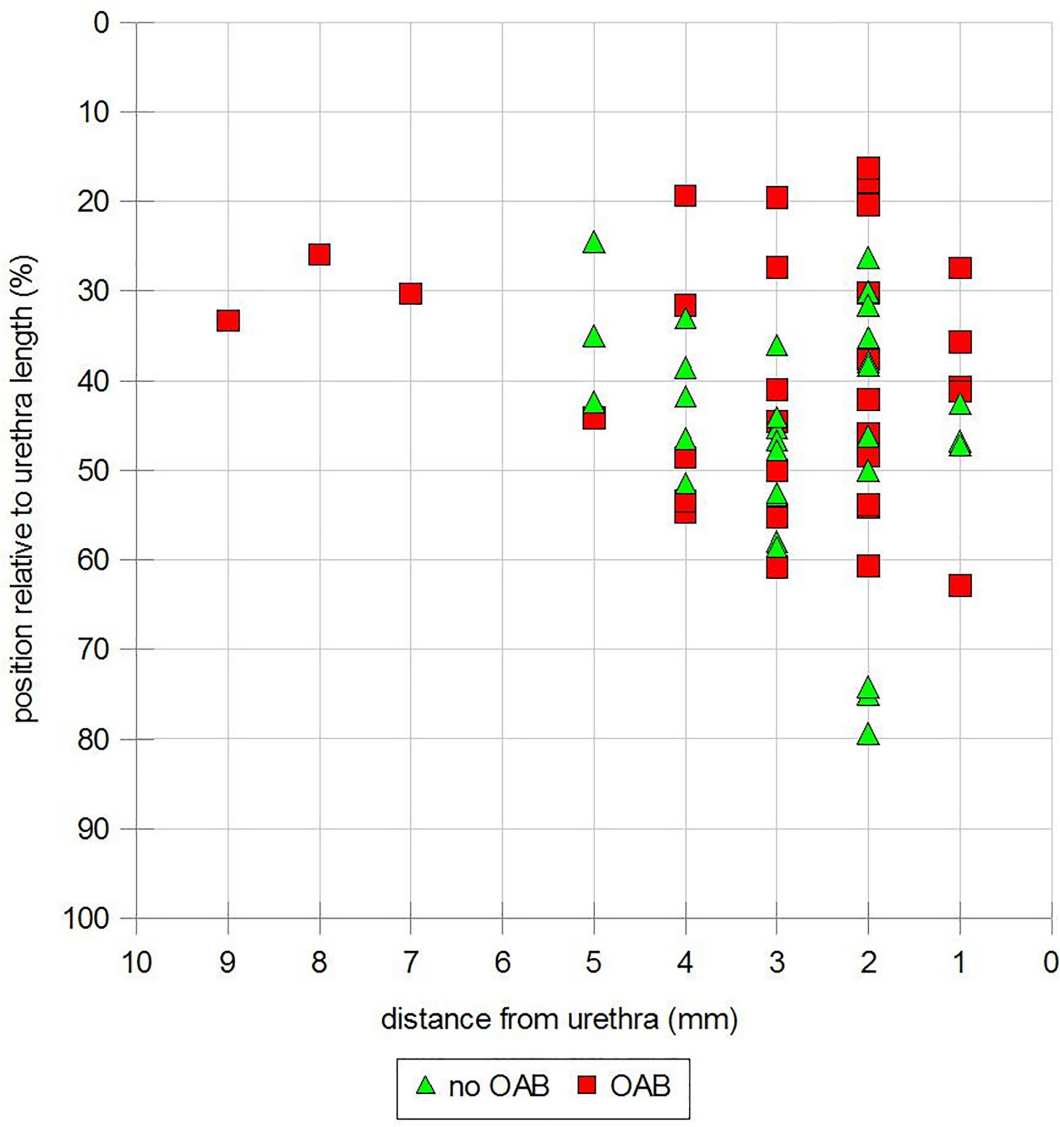
Outcome of women with or without OAB symptoms dependent on tape position relative to TUD and TP.

Preoperatively 20/67 women (29%) reported OAB symptoms and 3 of these (15%) had symptom resolution at follow-up. Fifteen women (22%) reported de novo OAB symptoms (McNemar test, p < .001). 50% of the patients describing OAB symptoms postoperatively were subjectively cured in regard to SUI and 34% objectively, respectively. However, only 7/39 (19%) of women with ongoing or de novo OAB symptoms at follow-up were receiving treatment for OAB. We found no differences in ultrasound measurements between women with or without OAB symptoms as can be seen in Table 5 and Fig 5.

**Table 5:** Results of ultrasound examination at 10 year follow-up. Comparison of women with and with and without OAB symptoms (n = 67).

	No OAB	OAB	p- Value
BNTD rest	13.59 ± 4.41	13.12 ± 5.63	.665
BNTD Valsalva	9.87 ± 6.47	11.82 ± 7.07	.845
CCTD rest	10.48 ± 8.37	8.42 ± 9.63	.379
CCTD Valsalva	5.53 ± 12.35	3.46 ± 8.72	.664
TP rest	45.88 ± 12.69	39.85 ± 13.74	.170
TP Valsalva	41.34 ± 16.79	38.37 ± 16.12	.554
TUD rest	2.82 ± 1.08	2.98 ± 1.91	.615
TUD Valsalva	2.62 ± 1.04	2.55 ± 1.59	.549

Data are expressed as median ± standard deviation except for the p-values. BNTD, bladder neck tape distance; CCTD, cranio-caudal tape distance; OAB, over active bladder; TP, tape percentile; TUD, tape urethral

QoL and sexual health results are detailed in supplementary Tables 1–3. With the KHQ, women who were either subjectively or objectively cured showed better QoL, especially in the domains role and physical limitation due to SUI, and emotions (supplementary Table 1). Similar findings were observed for the extended QoL score of the IOQ (supplementary Table 2). Forty-four women completed the FSFI questionnaire (supplementary 1able 3). There were no significant differences between objectively and subjectively cured women in almost all parameters.

## Discussion

To our knowledge this is the first description of long-term ultrasound findings and their correlation with clinical outcomes after transobturator midurethral sling surgery. According to our data, the optimal tape position appears to be in the middle third of the urethra, with a maximal tape-to urethra distance of 5mm. Objective success was lower if the tape was closer to the bladder neck or further away from the urethra.

Other groups have used ultrasound to assess midurethral tapes and correlated sonographic measurements with clinical outcomes. Jiang et al. found higher rates of persistent SUI in patients with tapes closer to the bladder neck [23–25].

Based on the data from this study TP is a very relevant parameter compared to BNTD, as it reflects the individual position of the tape and not an absolute distance in patients with different urethra lengths. Our finding that the tape percentile in objectively cured patients is significantly higher (45%) compared to not cured patients (37%) is consistent with the previous theories and studies [26,27,28]. Based on the estimation of Westby the zone of maximal urethral pressure is between 53 and 72% of the functional urethral length [29]; the optimal position of the retropubic tape has been reported to be between 50–70% of the sonographic urethra length [26]. Viereck and coworkers described different optimal tape percentiles for retropubic and transobturator tapes, suggesting that the incision for the transobturator tape should be more proximal compared to retropubic route to achieve optimal outcome [30], but tape position in the proximal third of the urethra are associated with higher failure rates [31].

The significant difference not only for TP, but also for TUD, in cured vs. not cured patients stands in line with recent reports [32]. The previously reported range of optimal TUD (3-5mm) has been confirmed by our present study with the calculated cut-off of 5mm[16].

Ultrasound is useful in assessing patients with dysfunction of the lower urogenital tract and specifically in assessing patients before and after urogynecologic surgery [33]. Ultrasound is particularly useful for evaluating patients with persisting or recurrent incontinence, voiding difficulties or OAB symptoms after surgery [34]. In the present study we found that tapes were readily visualized at 10 years. In particular, tapes located >5mm from the urethra were associated with SUI. This information might be useful in planning further management of patients with persistent problems.

Regarding OAB, surgical outcome after suburethral tape varies from symptom resolution of preexisting symptoms to de novo OAB incontinence. It is known, that tape position to close to the urethra correlates with de novo OAB symptoms and voiding dysfunction [35]. However, we could not detect any differences in tape position in our cohort between women with or without OAB symptoms. Although prediction of change for preexisting OAB symptoms is difficult, OAB is relevant in preoperative counseling.

Strengths of the present study are the long-term follow-up and the comprehensive clinical evaluation including ultrasound measurements. Bias of clinical findings were minimized by the fact that the surgeons themselves did not perform the follow-up examination. Subjective data were collected using validated questionnaires. A limitation of the study is the lack of data from longitudinal follow up to answer questions about possible migration of the sling and change of clinical symptoms over time. Also, patients who had undergone reoperation especially for voiding dysfunction were excluded; information on these patients might provide more insight into the mechanisms of success and failure of midurethral tape surgery. This study looked only at one transobturator tape and the findings may not apply to other tapes, including retropubic tapes.

## Conclusion

Location of the tape is crucial for subjective and objective cure of SUI even 10 years after surgery. Optimal tape location lies in the midurethra with a maximum distance of 5 mm to the urethra.

## Supporting information

S1 Table Results of the Kings health questionnaire at 10 year follow-up. Comparison of subjectively and objectively cured women to non-cured women (n = 67).
Data are expressed as median ± standard deviation except for the p-values. Lower scores indicate better QoL.
(DOCX)

S2 Table Results of incontinence outcome questionnaire at 10 year follow-up. Comparison of subjectively and objectively cured women to non-cured women (n = 67).
Data are expressed as median ± standard deviation except for the p-values. QoL, quality of life; OAB, overactive bladder. Lower scores indicate worse treatment outcome.
(DOCX)

S3 Table Results of the Kings health questionnaire at 10 year follow-up. Comparison of subjectively and objectively cured women to non-cured women (n = 67).
Data are expressed as median ± standard deviation except for the p-values. Higher scores indicate better sexual function.
(DOCX)

## References

[1] Coyne KS, Sexton CC, Irwin DE, Kopp ZS, Kelleher CJ, Milsom I. The impact of overactive bladder, incontinence and other lower urinary tract symptoms on quality of life, work productivity, sexuality and emotional well-being in men and women: results from the EPIC study. *BJU Int*. 2008;101(11):1388–1395. 10.1111/j.1464-410X.2008.07601.x 18454794

[2] Novara G, Artibani W, Barber MD, Chapple CR, Costantini E, Ficarra V, et al Updated systematic review and meta-analysis of the comparative data on colposuspensions, pubovaginal slings, and midurethral tapes in the surgical treatment of female stress urinary incontinence. *Eur Urol*. 2010;58(2):218–238. 10.1016/j.eururo.2010.04.022 20434257

[3] Wu JM, Matthews CA, Conover MM, Pate V, Jonsson Funk M. Lifetime risk of stress urinary incontinence or pelvic organ prolapse surgery. *Obstet Gynecol*. 2014;123(6):1201–1206. 10.1097/AOG.0000000000000286 24807341 PMC4174312

[4] Oliphant SS, Jones KA, Wang L, Bunker CH, Lowder JL. Trends over time with commonly performed obstetric and gynecologic inpatient procedures. *Obstet Gynecol*. 2010;116(4):926–931. 10.1097/AOG.0b013e3181f38599 20859157 PMC3253706

[5] Labrie J, Berghmans BL, Fischer K, Milani AL, van der Wijk I, Smalbraak DJ, et al Surgery versus physiotherapy for stress urinary incontinence. *N Engl J Med*. 2013;369(12):1124–1133. 10.1056/NEJMoa1210627 24047061

[6] Aigmuller T, Tammaa A, Tamussino K, Hanzal E, Umek W, Kolle D, et al Retropubic vs. transobturator tension-free vaginal tape for female stress urinary incontinence: 3-month results of a randomized controlled trial. *Int Urogynecol J*. 2014;25(8):1023–1030. 10.1007/s00192-014-2384-z 24819327

[7] Ford AA, Rogerson L, Cody JD, Aluko P, Ogah JA. Mid-urethral sling operations for stress urinary incontinence in women. *The Cochrane database of systematic reviews*. 2017;7:Cd006375 10.1002/14651858.CD006375.pub4 28756647 PMC6483329

[8] Ulrich D, Tammaa A, Holbfer S, Trutnovsky G, Bjelic-Radisic V, Tamussino K, et al Ten-Year Followup after Tension-Free Vaginal Tape-Obturator Procedure for Stress Urinary Incontinence. *J Urol*. 2016;196(4):1201–1206. 10.1016/j.juro.2016.05.036 27181504

[9] Aigmueller T, Trutnovsky G, Tamussino K, Kargl J, Wittmann A, Surtov M, et al Ten-year follow-up after the tension-free vaginal tape procedure. *Am J Obstet Gynecol*. 2011;205(5):496 e1–5. 21944223 10.1016/j.ajog.2011.07.010

[10] Bjelic-Radisic V, Dorfer M, Greimel E, Frudinger A, Tamussino K, Winter R. Quality of life and continence 1 year after the tension-free vaginal tape operation. *Am J Obstet Gynecol*. 2006;195(6):1784–1788. 10.1016/j.ajog.2006.07.014 17132481

[11] Tammaa A, Aigmuller T, Hanzal E, Umek W, Kropshofer S, Lang PFJ, et al Retropubic versus transobturator tension-free vaginal tape (TVT vs TVT-O): Five-year results of the Austrian randomized trial. *Neurourol Urodyn*. 2017. 10.1002/nau.23298 28464312

[12] Tamussino KF, Hanzal E, Kolle D, Ralph G, Riss PA. Tension-free vaginal tape operation: results of the Austrian registry. *Obstet Gynecol*. 2001;98(5 Pt 1):732–736. 11704161 10.1016/s0029-7844(01)01565-4

[13] Tamussino K, Hanzal E, Kolle D, Tammaa A, Preyer O, Umek W, et al Transobturator tapes for stress urinary incontinence: Results of the Austrian registry. *Am J Obstet Gynecol*. 2007;197(6):634e1–5. 10.1016/j.ajog.2007.08.018 18060959

[14] Tunn R, Albrich S, Beilecke K, Kociszewski J, Lindig-Knopke C, Reisenauer C, et al Interdisciplinary S2k Guideline: Sonography in Urogynecology: Short Version—AWMF Registry Number: 015/055. *Geburtshilfe Frauenheilkd*. 2014;74(12):1093–1098. 10.1055/s-0034-1383044 25568465 PMC4275315

[15] Dietz HP, Barry C, Lim YN, Rane A. Two-dimensional and three-dimensional ultrasound imaging of suburethral slings. *Ultrasound Obstet Gynecol*. 2005;26(2):175–179. 10.1002/uog.1945 15988786

[16] Kociszewski J, Rautenberg O, Kolben S, Eberhard J, Hilgers R, Viereck V. Tape functionality: position, change in shape, and outcome after TVT procedure—mid-term results. *Int Urogynecol J*. 2010;21(7):795–800. 10.1007/s00192-010-1119-z 20204326 PMC2876268

[17] de Leval J. Novel surgical technique for the treatment of female stress urinary incontinence: transobturator vaginal tape inside-out. *Eur Urol*. 2003;44(6):724–730. 14644127 10.1016/j.eururo.2003.09.003

[18] Bjelic-Radisic V, Dorfer M, Tamussino K, Greimel E. Psychometric properties and validation of the German-language King's Health Questionnaire in women with stress urinary incontinence. *Neurourol Urodyn*. 2005;24(1):63–68. 15578627 10.1002/nau.20092

[19] Bjelic-Radisic V, Dorfer M, Tamussino K, Frudinger A, Kern P, Winter R, et al The Incontinence Outcome Questionnaire: an instrument for assessing patient-reported outcomes after surgery for stress urinary incontinence. *Int Urogynecol J*. 2007;18(10):1139–1149. 10.1007/s00192-007-0302-3 17308862

[20] Srikrishna S, Robinson D, Cardozo L. Validation of the Patient Global Impression of Improvement (PGI-I) for urogenital prolapse. *Int Urogynecol J*. 2010;21(5):523–528. 10.1007/s00192-009-1069-5 20013110

[21] Berner MM, Kriston L, Zahradnik HP, Härter M, Rohde A. Überprüfung der Gültigkeit und Zuverlässigkeit des deutschen Female Sexual Function Index (FSFI-d). *Geburtshilfe Frauenheilkd*. 2004;64(03):293–303.

[22] Shek KL, Chantarasorn V, Dietz HP. The urethral motion profile before and after suburethral sling placement. *J Urol*. 2010;183(4):1450–1454. 10.1016/j.juro.2009.12.028 20171657

[23] Jiang YH, Wang CC, Chuang FC, Ke QS, Kuo HC. Positioning of a suburethral sling at the bladder neck is associated with a higher recurrence rate of stress urinary incontinence. *J Ultrasound Med*. 2013;32(2):239–245. 23341378 10.7863/jum.2013.32.2.239

[24] Gilchrist AS, Rovner ES. Sling location in women with recurrent stress urinary incontinence following midurethral sling. *Urology*. 2012;79(1):76–79. 10.1016/j.urology.2011.08.009 22014964

[25] Yang JM, Yang SH, Huang WC, Tzeng CR. Correlation of tape location and tension with surgical outcome after transobturator suburethral tape procedures. *Ultrasound Obstet Gynecol*. 2012;39(4):458–465. 10.1002/uog.10086 21919102

[26] Kociszewski J, Rautenberg O, Kuszka A, Eberhard J, Hilgers R, Viereck V. Can we place tension-free vaginal tape where it should be? The one-third rule. *Ultrasound Obstet Gynecol*. 2012;39(2):210–214. 10.1002/uog.10050 21793084

[27] de Leval J, Bouffioux C, Penders L. [Therapy of incontinence by fixation of the vagina to a pyramidal flap. 200 cases]. *Acta Urol Belg*. 1984;52(2):286–290. 6720477

[28] Petros PE, Ulmsten UI. An integral theory of female urinary incontinence. Experimental and clinical considerations. *Acta Obstet Gynecol Scand Suppl*. 1990;153:7–31. 2093278 10.1111/j.1600-0412.1990.tb08027.x

[29] Westby M, Asmussen M, Ulmsten U. Location of maximum intraurethral pressure related to urogenital diaphragm in the female subject as studied by simultaneous urethrocystometry and voiding urethrocystography. *Am J Obstet Gynecol*. 1982;144(4):408–412. 6889814 10.1016/0002-9378(82)90245-9

[30] Viereck V, Kuszka A, Rautenberg O, Wlazlak E, Surkont G, Hilgers R, et al Do different vaginal tapes need different suburethral incisions? The one-half rule. *Neurourol Urodyn*. 2015;34(8):741–746. 10.1002/nau.22658 25176293

[31] Kociszewski J, Fabian G, Grothey S, Kuszka A, Zwierzchowska A, Majkusiak W, et al Are complications of stress urinary incontinence surgery procedures associated with the position of the sling? *Int J Urol*. 2017;24(2):145–150. 10.1111/iju.13262 27907976

[32] Wlazlak E, Viereck V, Kociszewski J, Kuszka A, Rautenberg O, Walser C, et al Role of intrinsic sphincter deficiency with and without urethral hypomobility on the outcome of tape insertion. *Neurourol Urodyn*. 2017;36(7):1910–1916. 10.1002/nau.23211 28139863

[33] Schuettoff S, Beyersdorff D, Gauruder-Burmester A, Tunn R. Visibility of the polypropylene tape after tension-free vaginal tape (TVT) procedure in women with stress urinary incontinence: comparison of introital ultrasound and magnetic resonance imaging in vitro and in vivo. *Ultrasound Obstet Gynecol*. 2006;27(6):687–692. 10.1002/uog.2781 16710883

[34] Kociszewski J, Kolben S, Barski D, Viereck V, Barcz E. Complications following Tension-Free Vaginal Tapes: Accurate Diagnosis and Complications Management. *BioMed research international*. 2015;(53)83–91. 10.1155/2015/538391 PMC4418011 25973423

[35] Kociszewski J, Rautenberg O, Perucchini D, Eberhard J, Geissbuhler V, Hilgers R, et al Tape functionality: sonographic tape characteristics and outcome after TVT incontinence surgery. *Neurourol Urodyn*. 2008;27(6):485–490. 10.1002/nau.20556 18288705

