# Correction: Sonographic sling position and cure rate 10-years after TVT- O procedure

**DOI:** 10.1371/journal.pone.0212597

**Published:** 2019-02-14

**Authors:** Ayman Tammaa, Vesna Bjelic-Radisic, Susanne Hölbfer, Gerda Trutnovsky, Karl Tamussino, Thomas Aigmüller, Daniela Ulrich

The first author’s name is spelled incorrectly. The correct name is: Ayman Tammaa. The correct citation is: Tammaa A, Bjelic-Radisic V, Hölbfer S, Trutnovsky G, Tamussino K, Aigmüller T, et al. (2019) Sonographic sling position and cure rate 10-years after TVT- O procedure. PLoS ONE 14(1): e0209668. https://doi.org/10.1371/journal.pone.0209668
