# Supplementary material for: Sonographic sling position and cure rate 10-years after TVT- O procedure
Source: PLoS One. 2019 Jan 7;14(1):e0209668. doi: 10.1371/journal.pone.0209668 (PMC6322787; doi:10.1371/journal.pone.0209668)
Supplement: S1 Table — Comparison of subjectively and objectively cured women to non-cured women (n = 67). Data are expressed as median ± standard deviation except for the p-values. Lower scores indicate better QoL. (DOCX) [file pone.0209668.s001.docx]

Supplementary table 1. Results of the Kings Health Questionnaire at 10 year follow-up.

Comparison of subjectively and objectively cured women to non-cured women (n=67).

|  | **Subjective cure** | |  | **Objective cure** | |  |
| --- | --- | --- | --- | --- | --- | --- |
|  | **Cured** | **Not cured** | **p- Value** | **Cured** | **Not cured** | **p- Value** |
| General health perception | 36.31 ± 27.16 | 40.28 ± 15.19 | .373 | 37.76 ± 25.58 | 36.36 ± 17.19 | .951 |
| LUTS impact | 41.46 ± 32.30 | 59.26 ± 35.34 | .064 | 43.75 ± 33.09 | 60.61 ± 35.96 | .129 |
| Role limitation | 20.94 ± 28.80 | 55.88 ± 35.33 | .001 | 24.81 ± 31.51 | 59.09 ± 34.45 | .004 |
| Physical limitations | 24.58 ± 29.23 | 53.92 ± 40.62 | .010 | 29.43 ± 33.55 | 51.67 ± 39.64 | .082 |
| Social limitations | 5.56 ± 11.25 | 29.01 ± 35.78 | .012 | 10.07 ± 18.54 | 24.24 ± 39.70 | .496 |
| Personal relationship | 15.91 ± 31.90 | 47.22 ± 52.09 | .176 | 18.75 ± 35.21 | 45.83 ± 53.36 | .321 |
| Emotions | 14.67 ± 21.65 | 31.48 ± 27.55 | .015 | 17.65 ± 24.43 | 28.70 ± 24.83 | .074 |
| Sleep/energy | 27.19 ± 26.12 | 38.24 ± 28.73 | .166 | 27.65 ± 27.83 | 42.42 ± 21.56 | .046 |
| Severity measures | 48.36 ± 31.30 | 66.32 ± 29.85 | .051 | 51.23 ± 33.04 | 65.56 ± 23.76 | .209 |
| Overactive bladder | 46.99 ± 32.77 | 61.34 ± 36.63 | .157 | 48.64 ± 34.08 | 64.02 ± 34.67 | .191 |
| Leakage during activity | 55.56 ± 46.68 | 55.88 ± 42.87 | .979 | 51.47 ± 45.20 | 70.00 ± 42.16 | .251 |
| Enuresis | 90.63 ± 27.20 | 71.43 ± 48.80 | .311 | 86.11 ± 33.46 | 80.00 ± 44.72 | .821 |
| Leakage during intercourse | 83.33 ± 38.35 | 50.00 ± 57.74 | .160 | 73.68 ± 45.24 | 100.00 ± 0.00 | .323 |
| Recurrent LUT-infection | 75.00 ± 40.09 | 41.67 ± 49.16 | .101 | 69.23 ± 42.61 | 50.00 ± 70.71 | .609 |
| Painful bladder | 66.67 ± 45.37 | 63.64 ± 45.23 | .818 | 64.58 ± 45.39 | 70.00 ± 44.72 | .844 |
| Voiding difficulties | 62.50 ± 45.52 | 63.64 ± 39.31 | .946 | 62.96 ± 42.95 | 62.50 ± 47.87 | .974 |

Data are expressed as median ± standard deviation except for the p-values. Lower scores indicate better QoL
