# Supplementary material for: Sonographic sling position and cure rate 10-years after TVT- O procedure
Source: PLoS One. 2019 Jan 7;14(1):e0209668. doi: 10.1371/journal.pone.0209668 (PMC6322787; doi:10.1371/journal.pone.0209668)
Supplement: S2 Table — Comparison of subjectively and objectively cured women to non-cured women (n = 67). Data are expressed as median ± standard deviation except for the p-values. QoL, quality of life; OAB, overactive bladder. Lower scores indicate worse treatment outcome. (DOCX) [file pone.0209668.s002.docx]

Supplementary table 2. Results of Incontinence Outcome Questionnaire at 10 year follow-up. Comparison of subjectively and objectively cured women to non-cured women (n=67).

|  | **Subjective cure** | |  | **Objective cure** | |  |
| --- | --- | --- | --- | --- | --- | --- |
|  | **Cured** | **Not cured** | **p- Value** | **Cured** | **Not cured** | **p- Value** |
| Extended score (QoL, satisfaction) | 29.82 ± 16.34 | 45.57 ± 13.94 | .001 | 31.10 ± 16.57 | 47.32 ± 13.16 | .003 |
| Pain | 8.57 ± 17.19 | 15.00 ± 22.51 | .268 | 9.13 ± 18.24 | 15.00 ± 21.11 | .251 |
| Urinary infection | 23.81 ± 43.11 | 47.06 ± 51.45 | .082 | 25.53 ± 44.08 | 50.00 ± 52.22 | .103 |
| Other infection | 26.83 ± 44.86 | 52.94 ± 51.45 | .059 | 30.43 ± 46.52 | 50.00 ± 52.22 | .208 |
| Hospital readmission | 5.00 ± 22.07 | 5.56 ± 23.57 | .930 | 6.38 ± 24.71 | 0.00 ± 0.00 | .394 |
| Symptoms preoperative | 71.43 ± 28.99 | 73.61 ± 18.13 | .832 | 72.92 ± 27.21 | 68.75 ± 21.65 | .414 |
| OAB preoperative | 75.00 ± 43.92 | 77.78 ± 42.78 | .824 | 80.95 ± 39.74 | 58.33 ± 51.49 | .109 |

Data are expressed as median ± standard deviation except for the p-values. QoL, quality of life; OAB, overactive bladder. Lower scores indicate worse treatment outcome.
