# Supplementary material for: Sonographic sling position and cure rate 10-years after TVT- O procedure
Source: PLoS One. 2019 Jan 7;14(1):e0209668. doi: 10.1371/journal.pone.0209668 (PMC6322787; doi:10.1371/journal.pone.0209668)
Supplement: S3 Table — Comparison of subjectively and objectively cured women to non-cured women (n = 67). Data are expressed as median ± standard deviation except for the p-values. Higher scores indicate better sexual function. (DOCX) [file pone.0209668.s003.docx]

Supplementary table 3. Results of the FSFI-Questionnaire10 years after TVT-O. Comparison of subjectively and objectively

cured women to non-cured women (n=44).

|  | **Subjective cure** | |  | **Objective cure** | |  |
| --- | --- | --- | --- | --- | --- | --- |
|  | **Cured** | **Not cured** | **p- Value** | **Cured** | **Not cured** | **p- Value** |
| Desire | 2.30 ± 1.35 | 1.89 ± 1.07 | .309 | 2.24 ± 1.30 | 1.96 ± 1.20 | .380 |
| Arousal | 1.75 ± 1.84 | 0.98 ± 1.15 | .211 | 1.58 ± 1.80 | 1.23 ± 1.25 | .753 |
| Lubrication | 1.55 ± 2.07 | 0.42 ± 0.95 | .042 | 1.38 ± 2.01 | 0.57 ± 1.09 | .246 |
| Orgasm | 1.77 ± 2.25 | 0.64 ± 1.32 | .060 | 1.57 ± 2.19 | 0.87 ± 1.49 | .301 |
| Satisfaction | 2.02 ± 2.12 | 0.87 ± 1.28 | .079 | 1.81 ± 2.07 | 1.11 ± 1.42 | .368 |
| Pain | 2.32 ± 2.67 | 0.72 ± 1.85 | .082 | 2.04 ± 2.62 | 0.98 ± 2.12 | .369 |
| Full scale | 12.35 ± 11.01 | 5.77 ± 6.20 | .097 | 11.37 ± 10.87 | 6.73 ± 6.74 | .257 |

Data are expressed as median ± standard deviation except for the p-values. Higher scores indicate better sexual function
